# A genetic risk score is differentially associated with migraine with and without aura

**DOI:** 10.1007/s00439-017-1816-5

**Published:** 2017-06-27

**Authors:** Claudia Pisanu, Martin Preisig, Enrique Castelao, Jennifer Glaus, Giorgio Pistis, Alessio Squassina, Maria Del Zompo, Kathleen R. Merikangas, Gérard Waeber, Peter Vollenweider, Jessica Mwinyi, Helgi B. Schiöth

**Affiliations:** 1 Division of Functional Pharmacology, Department of Neuroscience, BMC, University of Uppsala, Husargatan 3, 75124 Uppsala, Sweden; 2 Department of Biomedical Sciences, University of Cagliari, Cagliari, Italy; 3 Department of Psychiatry, Lausanne University Hospital, Prilly, Switzerland; 4 Genetic Epidemiology Research Branch, Intramural Research Program, National Institute of Mental Health, Bethesda, MD USA; 5 Department of Internal Medicine, Lausanne University Hospital, Lausanne, Switzerland

## Abstract

Although a number of migraine-associated single-nucleotide polymorphisms (SNP) with small effect size have been identified, little is known about the additive impact of these variants on migraine risk, frequency and severity. We investigated to what extent a genetic risk score (GRS) based on recently published, novel migraine-associated SNPs is associated with migraine prevalence, subtypes and severity in a large population-based sample. The sample comprised 446 subjects with migraine and 2511 controls from the CoLaus/PsyCoLaus study. Fifty-four SNPs earlier associated with migraine were selected. SNPs with a low impact on migraine prevalence in our sample were excluded using random forest. We combined the remaining 21 SNPs into a GRS and analyzed the association with migraine using logistic regression models. The GRS was significantly associated with migraine (OR = 1.56, *p* = 0.02) and migraine without aura (MWOA) (OR = 2.01, *p* = 0.003), but not with migraine with aura (MWA). The GRS was not associated with migraine frequency, intensity or interference with daily activities. We show that a GRS combining multiple genetic risk variants is associated with MWOA but not MWA, suggesting a different genetic susceptibility background underlying the two forms of migraine.

**Electronic supplementary material:**

The online version of this article (doi:10.1007/s00439-017-1816-5) contains supplementary material, which is available to authorized users.

## Introduction

Migraine is a disabling disorder characterized by recurrent episodes of headache attacks and associated symptoms. With a lifetime prevalence of 18% in women and 6% in men, migraine is an extremely common disorder (GBD 2015 Disease and Injury Incidence and Prevalence Collaborators 2016). Being ranked as the seventh most disabling disease in terms of years lived with disability, migraine has also a substantial socioeconomic impact (GBD 2015 Disease and Injury Incidence and Prevalence Collaborators 2016).

Migraine without aura (MWOA) is the most common form of migraine and is characterized by recurrent severe headache attacks lasting 4–72 h with associated gastrointestinal and autonomic symptoms [Headache Classification Committee of the International Headache Society (IHS) 2013; Headache Classification Subcommittee of the International Headache Society 2004]. Up to one-third of affected individuals experience transient focal neurological symptoms before or during the headache attack [(migraine with aura (MWA)] [Headache Classification Committee of the International Headache Society (IHS) 2013; Headache Classification Subcommittee of the International Headache 2004]. Although a number of hypotheses have been advanced, the pathogenesis of migraine is hitherto not fully understood, which is, however, of urgent need for the development of novel treatment approaches.

Migraine is thought to be the result of both genetic and environmental factors. The heritability of migraine has been estimated at 50% (Mulder et al. 2003) and has been suggested to be different for the two subtypes. While first-degree relatives of patients with MWOA show a 1.9 times higher risk for MWOA and a 1.4 times higher risk for MWA, relatives of individuals with MWA have a 4 times higher risk to develop MWA while the risk to develop MWOA is not elevated (Silberstein and Dodick 2013). In recent years, several single-nucleotide polymorphisms (SNP) significantly associated with migraine have been identified (Anttila et al. 2010, 2013; Chasman et al. 2011, 2014 Freilinger et al. 2012; Gormley et al. 2016). A recent meta-analysis pooling data from 29 genome-wide association studies (GWAS) identified 12 SNPs associated with increased migraine risk and, specifically, with larger effect sizes in MWOA compared to MWA cases (Anttila et al. 2013). However, other studies suggested an overlap between genetic variants associated with the two subtypes (Nyholt et al. 2015; Zhao et al. 2016).

The largest meta-analysis conducted so far on migraine genetics has just been recently published and included data of 59,674 migraine subjects and 316,078 controls. Besides the replication of some of the previously associated loci, this study identified 34 novel migraine-associated SNPs (Gormley et al. 2016). Notably, while this meta-analysis identified seven loci specifically associated with MWOA, a subset analysis revealed no significant association for MWA (Gormley et al. 2016). Therefore, the question to what extent a genetic heterogeneity exists between MWOA and MWA remains open. Importantly, all identified migraine-associated SNPs show small effect sizes and account for a small part of migraine heritability (Gormley et al. 2016). Thus, the application of genetic risk score (GRS) analysis, which sums risk alleles across multiple genetic loci, may be of essential help to more efficiently explain the missing heritability of complex phenotypes, as seen in migraine. Recently, a GRS study suggested distinct genetic signatures for migraine with and without comorbid major depressive disorder (MDD) (Ligthart et al. 2014). Other studies investigated the genetic overlap between migraine and ischemic stroke (Malik et al. 2015) or coronary artery disease (Winsvold et al. 2015), finding a much stronger overlap between cardiovascular diseases and MWOA rather than MWA.

Although first studies started to elucidate the isolated and combined effect of genetic variants on migraine risk, the aspect to what extent genetic susceptibility markers may predispose to a more severe form of migraine has only been scarcely investigated. To date, only one study tested the association between a cumulative GRS composed of seven SNPs and four proxies for severe migraine traits, i.e., early onset, >100 lifetime migraine attacks, prolonged migraine attacks and migraine chronification, in a clinical sample comprising 1806 migraine patients (Esserlind et al. 2016). Two SNPs, rs2274316 (*MEF2D*) and rs11172113 (*LRP1*), showed a significant association with high migraine attack frequency. However, the analysis evaluating the association between the GRS and “many lifetime attacks” or “prolonged migraine attacks” only showed a non-significant trend.

A cumulative genetic risk evaluation of migraine subtypes, i.e., MWA and MWOA, and additional important aspects of migraine severity, including associated disabling symptoms, migraine intensity, and interference with daily activities under consideration of very recently detected novel migraine-associated variants would add further understanding to the genotype–phenotype interplay in migraine. To close this gap of knowledge, we investigated the association between genetic susceptibility factors, taking recently described, novel genetic susceptibility markers into consideration, and migraine prevalence and severity in a large population-based cohort. In the current study, we aimed to specifically evaluate if (1) a GRS based on migraine-associated SNPs is associated with migraine and migraine-type prevalence in this large population-based sample and if (2) the GRS is associated with migraine frequency and distinct traits of migraine severity.

## Methods

### Sample

The present study included data from the CoLaus|PsyCoLaus study (Firmann et al. 2008; Preisig et al. 2009), a population-based cohort study designed to investigate the genetic and environmental determinants of mental disorders and cardiovascular diseases (CVD) in the general population. Anthropometric measures, DNA and plasma samples were collected from a total of 6733 individuals (CoLaus), aged between 35 and 75 years, who were randomly selected between 2003 and 2006 according to the civil register of the city of Lausanne (Firmann et al. 2008). Sixty-seven percent of the participants of CoLaus in the age range of 35–66 years (*N* = 5535) also agreed to take part in the psychiatric evaluation (PsyCoLaus), resulting in a sample of 3719 individuals with both physical and psychiatric examination results (Preisig et al. 2009). The gender distribution of the PsyCoLaus sample (47% men) was similar compared to that of the general population in the same age range (Preisig et al. 2009). Scores on the General Health Questionnaire (GHQ-12) (Goldberg 1972), French translation (Bettschart and Bolognini 1996), a self-rating instrument completed at the somatic examination were similar for subjects who accepted to participate in PsyCoLaus and those who refused to participate. For the present analyses, we excluded 756 individuals because of missing genotyping data and 6 subjects because of missing lifetime MDD data, leaving a total of 2957 subjects. The CoLaus|PsyCoLaus was approved by the Institutional Ethics Committee of the University of Lausanne. Informed written consent was obtained from all participants.

### Assessment of clinical and biological data

The lifetime prevalence of migraine was assessed according to the criteria of the International Classification of Headache Disorders (ICHD-II), using the validated French version of the Diagnostic Interview for Headache Syndromes (DIHS). For all the participants who reported headache symptoms in the validated French translation (Leboyer et al. 1995) of the Diagnostic Interview for Genetic Studies (DIGS) (Nurnberger et al. 1994), these symptoms were examined in detail using the DIHS interview. Subjects that satisfied the ICHD-II criteria were considered to be affected by migraine and were compared with subjects who did not meet the criteria o who did not report headache symptoms. The lifetime prevalence of MDD was assessed using the validated French translation (Leboyer et al. 1995) of the DIGS (Nurnberger et al. 1994). The French translation of the DIGS revealed excellent inter-rater reliability in terms of kappa and Yul’’s *Y* coefficients for major mood and psychotic disorders and a slightly lower 6-week test–retest reliability (Preisig et al. 1999). Interviews were conducted by trained psychologists or psychiatrists and reviewed by an experienced senior psychologist. For each subject with a diagnosis of migraine, the following variables were extracted from the interview: age at onset, intensity of migraine (coded as a dichotomous variable comparing light/moderate versus severe migraine), migraine-associated gastrointestinal symptoms (loss of appetite, hunger, nausea, vomit, diarrhea and abdominal pain), duration of the interval between two attacks, duration of the attack, and interference with daily activities (evaluated with a score from 0 to 100).

### Genotyping

Nuclear DNA was extracted from whole blood of all participants included in the CoLaus study. Genome-wide genotyping was performed using Affymetrix 500K SNP chips. Subjects were excluded from the analysis in case of inconsistency between sex and genetic data, a genotype call rate less than 90%, or inconsistencies of genotyping results in duplicate samples. Quality control for SNPs was performed using the following criteria: SNPs monomorphic among all samples; SNPs with call rates less than 95% deviation from the Hardy–Weinberg equilibrium (HWE) (*p* < 1 × 10E−07). Imputation was performed using minimac (http://genome.sph.umich.edu/wiki/Minimac) and 1000 Genomes (phase 1, version 3).

Fifty-four SNPs were chosen among previously genome-wide determined, significant (*p* < 5 × 10E−08) migraine-associated SNPs for which an odds ratio (OR) was reported by previous studies (Anttila et al. 2010, 2013; Freilinger et al. 2012; Gormley et al. 2016) (Supplementary Table 1). For 51 of these SNPs genotype data were available in our sample (Table 1). In case that an SNP was imputed, an imputation quality (*r* ^2^−hat) >0.4 was set as threshold for the inclusion of the variant into further analyses.

**Table 1:** Demographic and clinical characteristics of the sample (*n* = 2957)

	Migraine vs. controls					MWA vs. controls			MWOA vs. controls	
	Controls	Migraine	Statistics	*p*	MWA	Statistics	*p*	MWOA	Statistics	*p*
*N*	2511	446	**–**	**–**	152		–	294		**–**
Age (years)	51.7 (±8.9)	50.6 (±8.6)	**519,856**	**0.016**	51.1 (±9.0)	183,737	0.44	48.4 (±8.3)	**336,119**	**0.012**
Sex female (%)	50%	69%	**55.27**	**<0.001**	60%	**5.34**	**0.02**	74%	**60.47**	**<0.001**
Lifetime MDD (%)	41%	57%	**41.47**	**<0.001**	63%	**29.45**	**<0.001**	54%	**19.09**	**<0.001**

Continuous variables are expressed as mean ± standard deviation; *p* values are calculated using Mann–Whitney test or Pearson’s Chi-square test, comparing subjects with migraine or migraine subtypes vs. controls

Significant differences are indicated in bold

*MDD* major depressive disorder, *MWA* migraine with aura, *MWOA* migraine without aura

### Statistical analysis

Differences in continuous or categorical variables between subjects with migraine and controls were assessed using Mann–Whitney test or Pearson’s Chi-square test, respectively.

Linkage disequilibrium (LD) between markers was measured by the r-squared coefficient using Haploview (v. 4.2) (Barrett et al. 2005). SNPs with *r* ^2^ values larger than 0.8 were excluded. Deviation from HWE was tested using Chi-square test (*p* < 0.001). HWE *p* value threshold was corrected according to Bonferroni (*p* < 0.001, i.e., 0.05/51 tested genetic variants). SNPs with a minor allele frequency (MAF) <0.01 were also excluded.

Genotypes for 51 out of 54 SNPs previously associated with migraine and for which an OR had been reported were available in our dataset (Supplementary Table 1). Two SNPs were not in HWE and were excluded. After the removal of SNPs with too low imputation quality (*n* = 0), with MAF <0.01 (*n* = 0) and of SNPs being in LD (*n* = 9), we proceeded with 40 SNPs in subsequent analyses.

The association between individual SNPs and migraine was tested using logistic regression analyses adjusting for age, sex and a lifetime diagnosis of MDD. A Bonferroni-adjusted *p* value of 0.0013 (0.05/40) was applied.

Analyses were performed applying an additive genetic model. The genotype of each SNP was coded based on the amount of effect alleles, i.e., 0 for no effect alleles, 1 for heterozygous SNP carriers and 2 for individuals carrying two effect alleles. Subsequently, SNP-associated beta coefficients were calculated based on the ORs reported in Anttila et al. 2013, Freilinger et al. 2012 and Gormley et al. 2016, and multiplied with the amount of alleles to obtain weighted SNP scores. A random forest model was constructed including weighted scores as predictors and a lifetime diagnosis of migraine as the target variable. The dataset was randomly partitioned to a training (70%), validation (15%) and testing (15%) dataset to obtain a better unbiased estimate of error. A total of 10,000 random decision trees were created. SNPs with a positive mean decrease accuracy (i.e., variables that cause subjects to be incorrectly classified if removed) were considered to have a relevant influence on the model and were chosen for inclusion in the GRS.

GRS was calculated by summing up the weighted SNP scores according to the following formula:

$$\mathop \sum \limits_{i = 1}^{n} k \,{\text{effect alleles}}_{{{\text{SNP}}i}} \times {\text{beta value}}_{{{\text{SNP}}i}}$$

with *n* the number of included SNPs in the model, and *k* the number of effect alleles. The load of effect alleles between subjects with migraine or migraine subtypes and controls was compared using the Mann–Whitney test.

The GRS was used as a continuous predictor in a binary logistic regression model with migraine (or migraine subtypes) as the dependent variable, adjusting for age, sex, and a lifetime diagnosis of MDD. The GRS was also coded as a dichotomous variable to compare the association between migraine and the upper/lower quartiles of the GRS distribution using a binary logistic regression model.

In a second step, we ran a case-only analysis to test the association between the GRS and characteristics of migraine severity. Association between GRS and age at onset, migraine frequency, duration of attacks, or interference with daily activities was tested performing Spearman’s correlation analyses, while the association between GRS and migraine intensity or gastrointestinal symptoms was tested with the Mann–Whitney test. To test the association between the GRS and continuous or dichotomous clinical characteristics of migraine, we constructed linear or logistic regression models, respectively, adjusting for age, sex and lifetime MDD. A *p* value <0.05 was considered significant. Analyses were performed using Haploview, the package Rattle (Graham 2011) in R (v. 3.2.3), SPSS v. 21 (IBM, Armonk, NY, USA), and GraphPad Prism 5.

## Results

### Demographic and clinical characteristics of the sample

The demographic and clinical characteristics of the sample are shown in Table 1. Within the sample of 2957 participants, 446 subjects (15%) met the lifetime diagnostic criteria for migraine. Among them, 34% fulfilled the criteria for MWA. Subjects with migraine were more likely to be younger, female, and to have a higher lifetime prevalence of MDD (Table 1). Therefore, analyses were adjusted for these variables.

### Isolated SNPs have only a small impact on migraine risk

We first tested the association between migraine and the 40 genetic variants individually in unadjusted analyses applying an additive genetic model and using logistic regression analysis (Supplementary Table 2).

Unadjusted analyses showed that two SNPs were associated with migraine at a Bonferroni corrected threshold of 0.0013 (0.05/40): rs4814864, located in the *SLC24A3* gene (OR = 1.32, *p* = 0.0008), and rs186166891, located in the *SUGCT* gene (OR = 1.48, *p* = 0.0004). One genetic variant was associated with migraine at a nominal level (rs1024905, OR = 1.19, *p* = 0.016).

Subsequently, we applied a logistic regression analysis with migraine as the outcome variable, adjusting for age, sex, and a lifetime diagnosis of MDD. The association of rs186166891 with migraine was confirmed (OR = 1.54, *p* = 0.0002), while rs4814864 was associated with migraine only at a nominal level (OR = 1.30, *p* = 0.0017).

The SNP rs186166891 was associated with MWOA (unadjusted OR = 1.58, *p* = 0.0004; adjusted OR: 1.66, *p* = 0.0001), but not with MWA (adjusted OR: 1.32, *p* = 0.15).

Analyses conducted in migraine subtypes also showed an association between rs1024905 and MWOA (unadjusted OR = 1.41, *p* = 0.0001; adjusted OR = 1.42, *p* = 0.0001), and a nominal association between rs4814864 and MWOA (unadjusted OR = 1.27, *p* = 0.017). Only one SNP was nominally associated with MWA (rs4814864, unadjusted OR = 1.41, *p* = 0.008; adjusted OR = 1.37, *p* = 0.015). None of the other variants was associated with migraine or migraine subtypes. These results pointed to a relatively small impact of single SNPs on migraine and migraine subtypes in our population-based cohort.

### A polygenic risk score composed of susceptibility SNPs for migraine is significantly associated with MWOA

**Fig. 1:**
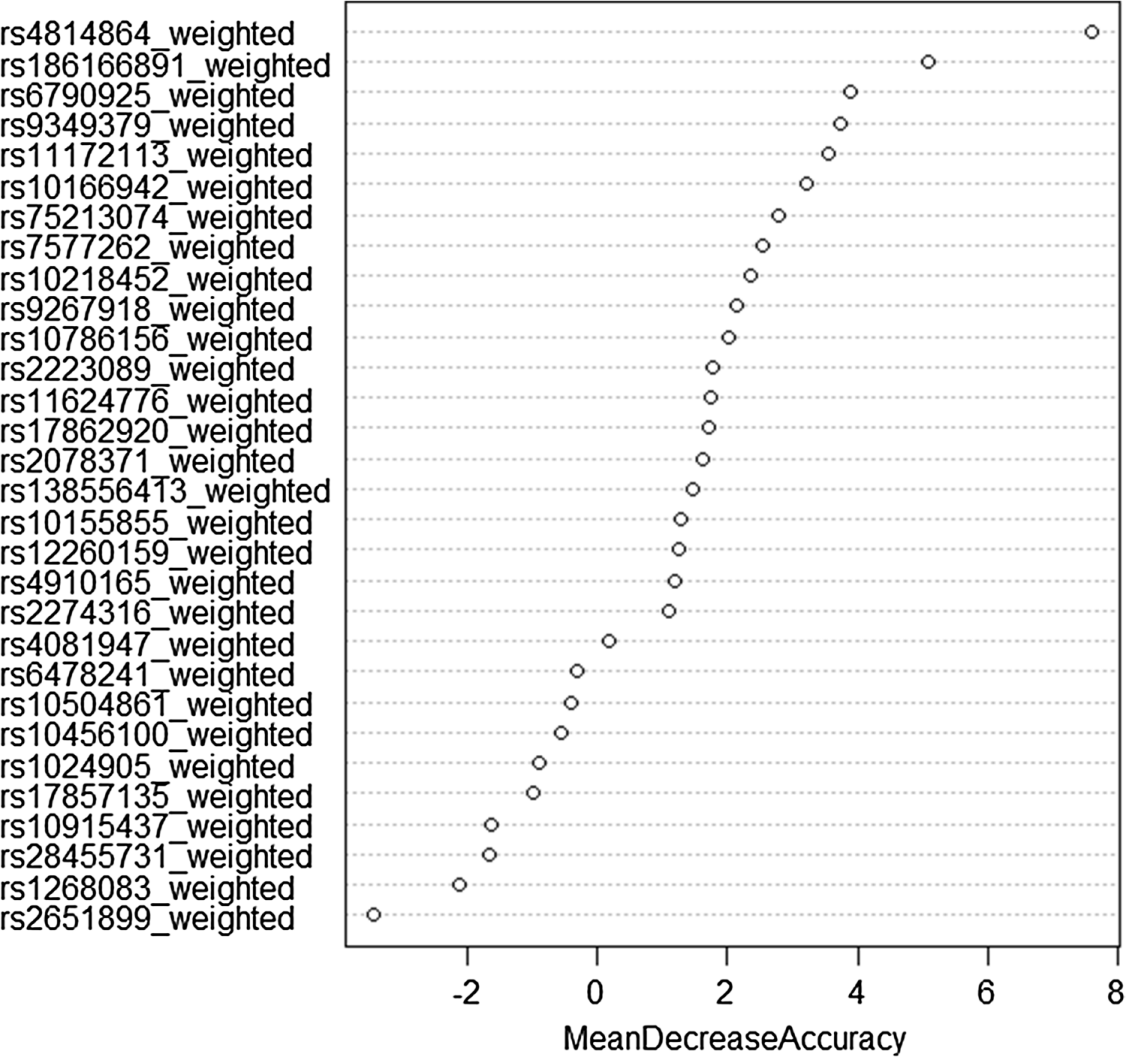
Random forest model including 21 SNPs previously associated with migraine. The first 30 variables with the highest mean decrease accuracy are plotted. Twenty-one migraine-associated SNP were shown to induce a positive change in mean decrease accuracy. These SNPs were thus considered to have a relevant influence on the model and were chosen for inclusion in the GRS. *GRS* genetic risk score, *SNP* single-nucleotide polymorphism

To weight the genetic variants with regard to their impact on migraine outcome, a random forest algorithm was developed. As shown in Table 2 and Fig. 1, a total of 21 genetic variants (closest genes: *MEF2D, PRDM16, TSPAN2, TRPM8, CARF, TGFBR2, PHACTR1, NOTCH4, SUGCT, DOCK4, PLCE1, HPSE2, ARMS2, MRVI1, LRP1, ITPK1, ZCCHC14, NLRP1*, and *SLC24A3*) induced a positive mean decrease accuracy and were considered in the subsequent calculation of the weighted GRS. The study participants showed a GRS distribution ranging from −1.33 to 0.76.

**Table 2:** SNPs previously associated with migraine and included in the GRS

SNP	Chr	Location	Gene	MA	MAF	Reported OR (95% CI)	Beta coefficient^b^	References
rs2274316	1	Genic	*MEF2D*	C	0.37	1.07 (1.04**–**1.09)	0.07	Anttila et al. 2013
rs10218452	1	Genic	*PRDM16*	G	0.22	1.11 (1.10**–**1.13)	0.10	Gormley et al. 2016
rs2078371	1	Intergenic	Near *TSPAN2*	C	0.12	1.11 (1.09**–**1.13)	0.10	Gormley et al. 2016
rs7577262	2	Genic	*TRPM8*	A	0.10	0.87 (0.84**–**0.90)	−0.14	Anttila et al. 2013
rs17862920	2	Genic	*TRPM8*	T	0.10	0.77 (0.70**–**0.84)	−0.26	Freilinger et al. 2012
rs10166942	2	Intergenic	Near *TRPM8*	C	0.20	0.94 (0.89**–**0.99)	−0.06	Gormley et al. 2016
rs138556413	2	Genic	*CARF*	T	0.03	0.88 (0.84**–**0.92)	−0.13	Gormley et al. 2016
rs6790925	3	Intergenic	Near *TGFBR2*	T	0.38	1.15 (1.10**–**1.21)	0.14	Anttila et al. 2013
rs9349379	6	Genic	*PHACTR1*	G	0.41	0.93 (0.92**–**0.95)	−0.07	Gormley et al. 2016
rs9267918^a^	6	Intergenic	Near *NOTCH4*	A	0.06	0.91 (0.88**–**0.94)	−0.09	Gormley et al. 2016
rs186166891	7	Genic	*SUGCT*	T	0.11	1.09 (1.07**–**1.12)	0.09	Gormley et al. 2016
rs10155855	7	Intergenic	Near *DOCK4*	T	0.05	1.08 (1.05**–**1.12)	0.08	Gormley et al. 2016
rs10786156	10	Genic	*PLCE1*	G	0.45	0.95 (0.94**–**0.96)	−0.05	Gormley et al. 2016
rs12260159	10	Genic	*HPSE2*	A	0.07	0.92 (0.89**–**0.94)	−0.08	Gormley et al. 2016
rs2223089	10	Intergenic	Near *ARMS2*	C	0.08	0.93 (0.91**–**0.95)	−0.07	Gormley et al., 2016
rs4910165	11	Genic	*MRVI1*	C	0.33	0.94 (0.91**–**0.98)	−0.06	Gormley et al. 2016
rs11172113	12	Genic	*LRP1*	C	0.42	0.90 (0.89**–**0.91)	−0.11	Gormley et al. 2016
rs11624776	14	Intergenic	Near *ITPK1*	C	0.31	0.96 (0.94**–**0.97)	−0.04	Gormley et al. 2016
rs4081947	16	Intergenic	Near *ZCCHC14*	G	0.34	1.03 (1.00**–**1.06)	0.03	Gormley et al. 2016
rs75213074	17	Intergenic	Near *NLRP1*	T	0.03	0.89 (0.86**–**0.93)	−0.12	Gormley et al. 2016
rs4814864	20	Genic	*SLC24A3*	C	0.26	1.07 (1.06**–**1.09)	0.07	Gormley et al. 2016

^a^In the meta-analysis by Gormley et al. (2016), the association is reported for rs140002913 (now merged into rs9267918)

^b^The beta coefficients column shows natural logarithms of the ORs reported by previous studies

*Chr* chromosome, *CI* confidence interval, *MA* minor allele, *MAF* minor allele frequency, *OR* odds ratio, *SNP* single-nucleotide polymorphism

**Fig. 2:**
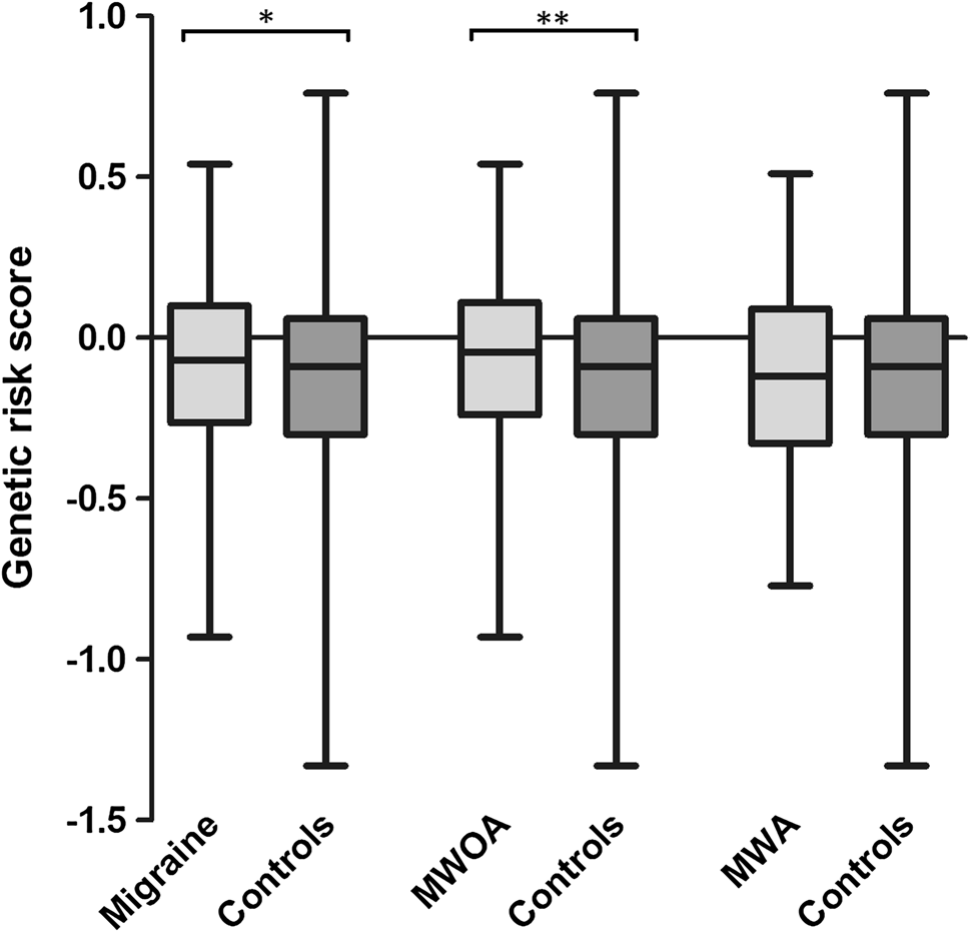
Box and whisker plot of GRS in subjects with migraine or migraine subtypes compared to controls. Mann–Whitney test showed a significant association between the genetic risk score and migraine (*U* = 522,400, *p* = 0.02) or MWOA (*U* = 329,900, *p* = 0.003), but not the MWA subtype (*U* = 189,200, *p* = 0.8). * <0.05, ** <0.005. *GRS* genetic risk score, *MWA* migraine with aura, *MWOA* migraine without aura

Mann–Whitney test showed that subjects with migraine or MWOA, but not MWA, had a higher load of risk alleles compared to controls (Fig. 2). As shown in Table 3, binary logistic regression analysis confirmed the significant impact of the GRS on the diagnosis of migraine. The covariates female sex, younger age, and a lifetime diagnosis of MDD were also significantly associated with a diagnosis of migraine.

**Table 3:** Association between GRS and diagnosis of migraine and migraine subtypes according to logistic regression models

	Migraine (*N* = 446)		MWA (*N* = 152)		MWOA (*N* = 294)	
	vs. controls (2511)					
	OR (95% CI)	*p*	OR (95% CI)	*p*	OR (95% CI)	*p*
GRS	**1.56 (1.07–2.27)**	**0.020**	0.96 (0.54–1.72)	0.890	**2.01 (1.28–3.17)**	**0.003**
Female sex	**2.04 (1.63–2.55)**	**<0.001**	1.22 (0.86–1.72)	0.260	**2.73 (2.06–3.61)**	**<0.001**
Age	**0.99 (0.97–1.00)**	**0.010**	1.00 (0.98–1.01)	0.590	**0.98 (0.97–0.99)**	**0.004**
MDD	**1.63 (1.32–2.01)**	**<0.001**	**2.37 (1.67–3.36)**	**<0.001**	**1.34 (1.04–1.72)**	**0.023**

Significant differences are indicated in bold

*CI* confidence interval, *GRS* genetic risk score, *MDD* major depressive disorder, *MWA* migraine with aura, *MWOA* migraine without aura, *OR* odds ratio

Importantly, when stratifying migraine according to the main clinical subtypes MWA and MWOA, only MWOA appeared to be significantly impacted by the GRS (Table 3). When the GRS was dichotomized to compare migraine prevalence between the bottom and top quartiles of the GRS distribution, the GRS was still associated with migraine [OR: 1.45 (95% confidence interval = 1.09–1.93), *p* = 0.011] and MWOA [OR: 1.76 (95% confidence interval = 1.24–2.50), *p* = 0.002] but not MWA (OR = 1.03, *p* = 0.89) in a regression logistic analysis adjusted for sex, age, and lifetime MDD.

### Association between GRS and clinical characteristics of migraine

We evaluated furthermore to what extent the GRS was associated with clinical features characterizing migraine severity, including migraine intensity, associated gastrointestinal symptoms, age at onset, interference with daily activities, and migraine duration and frequency. As shown in Table 4, the GRS was not significantly associated with any of the tested variables in the whole sample or in migraine subtypes.

**Table 4:** Association between GRS and clinical characteristics of migraine

	Migraine	Stat	p	Adj *p* ^a^	MWA	Stat	*p*	Adj *p* ^a^	MWOA	Stat	p	Adj *p* ^a^
	(*n* = 446)				(*n* = 152)				(*n* = 294)			
Age at onset (years)	24.41 (±12.35)	−0.036	0.45	0.66	23.66 (±11.64)	0.044	0.59	0.87	24.80 (±12.71)	−0.078	0.18	0.33
Gastrointestinal symptoms (%)	86.3%	11,710	0.97	0.74	82.9%	1394	0.23	0.29	88.1%	3940	0.21	0.17
Severe intensity (%)	76.0%	18,055	0.98	0.81	74.3%	2003	0.54	0.32	76.5%	7418	0.58	0.72
Interval between two attacks (days)	41.47 (±71.04)	0.001	0.98	0.79	60.34 (±105.00)	0.006	0.95	0.98	32.59 (±44.95)	0.014	0.83	0.43
Duration of the attack (h)	26.83 (±23.78)	0.008	0.87	0.12	22.05 (±23.79)	−0.028	0.73	0.16	29.23 (± 23.45)	−0.012	0.84	0.33
Interference with daily activities	71.38 (±30.15)	0.027	0.57	0.61	71.16 (±30.21)	−0.098	0.23	0.30	71.50 (±30.18)	0.092	0.12	0.14

Continuous variables are expressed as mean ± standard deviation

Unadjusted association between GRS and continuous (age at onset, interval between two attacks, duration of the attack, and interference with daily activities) or dichotomous variables (gastrointestinal symptoms and severe intensity) was tested using Spearman’s correlation test or Mann–Whitney test, respectively

*Adj* adjusted, *GRS* genetic risk score, *MWA* migraine with aura, *MWOA* migraine without aura, *Stat* statistics

^a^Adjusted association between GRS and continuous or dichotomous variables was tested using linear or logistic regression models, respectively, adjusting for age, sex, and lifetime diagnosis of major depressive disorder

## Discussion

The current study is, to our knowledge, the first to investigate the association between a polygenic risk score and migraine taking very recently described novel genetic susceptibility markers into account. Importantly, we investigate for the first time to what extent the developed GRS impacts the prevalence of migraine subtypes and migraine severity in a large population-based sample, adjusting for demographic and clinical confounders. We demonstrate that the GRS based on SNPs previously identified in GWAS studies or meta-analyses to be associated with migraine specifically impacts MWOA, but not the risk for MWA. These findings allow the suggestion that MWOA and MWA have a different genetic susceptibility background that plays a role in their pathogenesis.

First-degree relatives of individuals with MWA show a considerably higher risk of MWA (4 times) compared to the higher risk of MWOA shown by first-degree relatives of MWOA patients (1.9 times) (Silberstein and Dodick 2013). On the basis of these estimates, the genetic contribution to migraine pathogenesis has been hypothesized to be higher for MWA compared to MWOA. However, a recent meta-analysis of GWAS, including data from 23,285 migraine cases and 95,425 controls, failed to identify genetic variants significantly predisposing to MWA (Anttila et al. 2013). Since this meta-analysis only evaluated the association of common SNPs, a possible explanation for this inconsistency lays in the fact that genetic predisposition to MWA could be mediated by rare variants with large effect size (Anttila et al. 2013). A recent reanalysis of the same data found significant heterogeneity of SNP effects between MWA and MWOA, although the direction of effect for the majority of selected SNPs was found to be the same between the two subtypes (Nyholt et al. 2015). The largest meta-analysis conducted so far, including data from 59,674 affected subjects and 316,078 controls, also failed to identify SNPs associated with MWA (Gormley et al. 2016). Our results support the hypothesis that common variants with small effect sizes, even when evaluating them additively by GRS analysis, do not show an impact on MWA diagnosis.

Among the new susceptibility loci identified by Gormley et al. (Gormley et al. 2016) and included in our GRS, two lie in or near genes involved in regulation of vascular tone (*MEF2D, DOCK4,* and *SLC24A3*) (Dong et al. 2006; Firulli et al. 1996; Kang et al. 2015), while *ARMS2, PHACTR1, TGFBR2, LRP1,* and *NOTCH4* have been previously associated with vascular disease (Delev et al. 2017; Beaudoin et al. 2015; Guo et al. 2016; Hayashi et al. 2010; Yang et al. 2016), consistently with the suggested role of vascular dysfunction in migraine pathogenesis. Interestingly, one of the novel migraine-associated SNPs included in our GRS, rs75213074, is located near *NLRP1*, which encodes for the sensor component of an inflammasome (a protein complex that activates caspases and cytokines in response to damage-associated signals) (Guo et al. 2015). As outlined by a recent review, due to their ability to integrate various inflammatory signals and their expression in endothelial cells, neurons, and astrocytes, inflammasomes represent a promising functional link between vascular and neurological diseases (Lenart et al. 2016).

Similarly to other complex phenotypes, migraine heritability appears to be due to a large number of weak associations with many genetic variants, rather than few common risk variants (Anttila et al. 2013; Chasman et al. 2016). Therefore, a GRS evaluating the cumulative effect of multiple variants with a small impact is more likely to be able to explain a part of migraine missing heritability.

To date, only a few studies evaluated the association between a GRS and migraine prevalence in a population-based sample. Rodriguez-Acevedo and coworkers found an association between migraine and a GRS (including 51 genetic variants previously nominally associated with migraine) in a small sample including 74 cases and 211 controls (Rodriguez-Acevedo et al. 2015). We used a different approach by selecting only genetic variants previously associated with migraine with a genome-wide significant threshold for which an OR had been reported and we further prioritized variables using a random forest algorithm. Another study investigated the overlap between migraine and MDD, showing that the subgroup of patients with migraine and comorbid MDD are genetically most similar to MDD patients (Ligthart et al. 2014). On the basis of these results, it was suggested that migraine with and without underlying MDD might have different genetic substrates (Ligthart et al. 2014).

In our study, a large percentage of subjects with migraine showed underlying MDD, with subjects with MWA presenting a higher risk (OR = 2.36) compared with the MWOA subtype (OR = 1.35). It cannot be excluded that the specificity of our GRS for MWOA could be partly due to the lower prevalence of MDD in this subsample. It is possible that genetic variants with an overlapping impact on both migraine and MDD risk might exert a higher impact on the MWA subtype.

Only one study evaluated the association between a GRS and proxies of migraine severity in a clinical sample (Esserlind et al. 2016). The authors reported that a polygenic risk score including 12 loci was not associated with early onset, frequency, duration of attacks, and tendency to chronification. Our results confirm and extend these findings reporting no association between a GRS and other characteristics of migraine severity, such as migraine intensity, gastrointestinal-associated symptoms, or interference with daily activities.

Our results must be interpreted in light of their limitations. Our study was not powered enough to investigate the association between migraine and rare genetic variants, which might especially play a role in MWA. Since available studies, including the largest meta-analysis conducted to date (Gormley et al. 2016), have not been able to identify genetic variants significantly associated with MWA, further studies characterized by larger sample size and powered enough to identify rare genetic variants could be of help to construct a GRS that could prove to be more specific for MWA. However, it cannot be excluded that genetics might play a minor role in MWA pathogenesis and that other molecular biological mechanisms (e.g., methylation) may be of stronger relevance for MWA. Moreover, our study included a lower number of participants with MWA compared to MWOA, which could have limited our statistical power to identify a significant association between the GRS and this subtype. Future studies including a balanced number of participants with MWOA and MWA are needed to confirm our findings. Finally, for all the participants the interviews were conducted by trained psychologists or psychiatrists and reviewed by an experienced senior psychologist, but not by headache specialists.

## Conclusion

In conclusion, the present study suggests that a GRS combining the effect of multiple loci is associated with MWOA but not MWA in a large population-based sample. Genetic risk variants so far detected to play a role in migraine are not able to explain a comprehensive set of clinical characteristics of migraine severity.

## Electronic supplementary material

Below is the link to the electronic supplementary material.

Supplementary material 1 (DOCX 23 kb)

Supplementary material 2 (DOCX 26 kb)
