# Supplementary material for: A genetic risk score is differentially associated with migraine with and without aura
Source: Hum Genet. 2017 Jun 27;136(8):999–1008. doi: 10.1007/s00439-017-1816-5 (PMC5502071; doi:10.1007/s00439-017-1816-5)
Supplement: Supplementary file 1 — Supplementary material 1 (DOCX 23 kb) [file 439_2017_1816_MOESM1_ESM.docx]

**Supplementary table 1. SNPs previously associated with migraine at a genome-wide significant threshold for which a reported OR was available.**

| **SNP** | **Chr** | **Location** | **Gene** | **MA** | **MAF** | **Reported OR (95% CI)** | **Reference** | **Reason for exclusion from the GRS** |
| --- | --- | --- | --- | --- | --- | --- | --- | --- |
| rs2651899 | 1 | Genic | *PRDM16* | C | 0.41 | 1.09 (1.07-1.12) | Anttila et al., 2013 | Mean decrease accuracy < 0 |
| rs2274316 | 1 | Genic | *MEF2D* | C | 0.37 | 1.07 (1.04-1.09) | Anttila et al., 2013 | Included |
| rs10915437 | 1 | Intergenic | near *AJAP1* | G | 0.36 | 0.86 (0.82-0.91) | Anttila et al., 2013 | Mean decrease accuracy < 0 |
| rs10218452 | 1 | Genic | *PRDM16* | G | 0.22 | 1.11 (1.10-1.13) | Gormley et al., 2016 | Included |
| rs2078371 | 1 | Intergenic | near *TSPAN2* | C | 0.12 | 1.11 (1.09-1.13) | Gormley et al., 2016 | Included |
| rs1572668 | 1 | Intergenic | 1p31.1 | G | 0.48 | 1.04 (1.02-1.05) | Gormley et al., 2016 | Mean decrease accuracy < 0 |
| rs12134493 | 1 | Intergenic | near *TSPAN2* | A | 0.46 | 1.14 (1.10-1.18) | Anttila et al., 2013 | LD (r2 > 0.8) |
| rs1050316 | 1 | Genic | *MEF2D* | G | 0.34 | 1.24 (1.15-1.33) | Freilinger et al., 2012 | LD (r2 > 0.8) |
| rs3790455 | 1 | Genic | *MEF2D* | C | 0.34 | 1.24 (1.15-1.34) | Freilinger et al., 2012 | LD (r2 > 0.8) |
| rs3790459 | 1 | Genic | *MEF2D* | A | 0.35 | 1.24 (1.15-1.33) | Freilinger et al., 2012 | LD (r2 > 0.8) |
| rs12136856 | 1 | Genic | *MEF2D* | C | 0.34 | 1.23 (1.15-1.33) | Freilinger et al., 2012 | LD (r2 > 0.8) |
| rs1925950 | 1 | Genic | *MEF2D* | G | 0.35 | 1.07 (1.06-1.09) | Gormley et al., 2016 | LD (r2 > 0.8) |
| rs6693567 | 1 | Intergenic | near *ADAMTSL4* | C | 0.27 | 1.05 (1.03-1.06) | Gormley et al., 2016 | HWE (p < 0.0001) |
| rs7577262 | 2 | Genic | *TRPM8* | A | 0.10 | 0.87 (0.84-0.90) | Anttila et al., 2013 | Included |
| rs17862920 | 2 | Genic | *TRPM8* | T | 0.10 | 0.77 (0.70-0.84) | Freilinger et al., 2012 | Included |
| rs10166942 | 2 | Intergenic | near *TRPM8* | C | 0.20 | 0.94 (0.89-0.99) | Gormley et al., 2016 | Included |
| rs138556413 | 2 | Genic | *CARF* | T | 0.03 | 0.88 0.84-0.92) | Gormley et al., 2016 | Included |
| rs6790925 | 3 | Intergenic | near *TGFBR2* | T | 0.38 | 1.15 (1.10-1.21) | Anttila et al., 2013 | Included |
| rs13078967 | 3 | Intergenic | near *GPR149* | C | 0.03 | 0.87 (0.83-0.91) | Gormley et al., 2016 | Mean decrease accuracy < 0 |
| rs7640543 | 3 | Intergenic | near *TGFBR2* | A | 0.32 | 0.86 (0.81-0.91) | Freilinger et al., 2012 | LD (r2 > 0.8) |
| rs6791480 | 3 | Intergenic | near *TGFBR2* | T | 0.31 | 1.04 (1.03-1.06) | Gormley et al., 2016 | LD (r2 > 0.8) |
| rs7684253 | 4 | Intergenic | near *REST* | C | 0.45 | 0.96 (0.94-0.97) | Gormley et al., 2016 | Mean decrease accuracy < 0 |
| rs13208321 | 6 | Genic | *FHL5* | A | 0.22 | 1.18 (1.13-1.24) | Anttila et al., 2013 | Mean decrease accuracy < 0 |
| rs9349379 | 6 | Genic | *PHACTR1* | G | 0.41 | 0.93 (0.92-0.95) | Gormley et al., 2016 | Included |
| rs10456100 | 6 | Genic | *KCNK5* | T | 0.28 | 1.06 (1.04-1.07) | Gormley et al., 2016 | Mean decrease accuracy < 0 |
| rs1268083 | 6 | Genic | *LOC105377986* | C | 0.48 | 0.96 (0.95-0.97) | Gormley et al., 2016 | Mean decrease accuracy < 0 |
| rs28455731 | 6 | Intergenic | near *GJA1* | T | 0.16 | 1.06 (1.04-1.08) | Gormley et al., 2016 | Mean decrease accuracy < 0 |
| rs9267918* | 6 | Intergenic | near *NOTCH4* | A | 0.06 | 0.91 (0.88-0.94) | Gormley et al., 2016 | Included |
| rs67338227 | 6 | Genic | *FHL5* | T | 0.23 | 1.09 (1.08-1.11) | Gormley et al., 2016 | NA |
| rs186166891 | 7 | Genic | *SUGCT* | T | 0.11 | 1.09 (1.07-1.12) | Gormley et al., 2016 | Included |
| rs10155855 | 7 | Intergenic | near *DOCK4* | T | 0.05 | 1.08 (1.05-1.12) | Gormley et al., 2016 | Included |
| rs4379368 | 7 | Genic | *SUGCT* | T | 0.12 | 1.11 (1.08-1.15) | Anttila et al., 2013 | LD (r2 > 0.8) |
| rs10504861 | 8 | Intergenic | near *MMP16* | T | 0.16 | 0.86 (0.81-0.90) | Anttila et al., 2013 | Mean decrease accuracy < 0 |
| rs1835740 | 8 | Genic | *LOC105375655* | A | 0.35 | 1.18 (1.13-1.24) | Anttila et al., 2010 | HWE (p < 0.0001) |
| rs6478241 | 9 | Genic | *ASTN2* | A | 0.36 | 1.05 (1.04-1.07) | Gormley et al., 2016 | Mean decrease accuracy < 0 |
| rs10786156 | 10 | Genic | *PLCE1* | G | 0.45 | 0.95 (0.94-0.96) | Gormley et al., 2016 | Included |
| rs12260159 | 10 | Genic | *HPSE2* | A | 0.07 | 0.92 (0.89-0.94) | Gormley et al., 2016 | Included |
| rs2506142 | 10 | Genic | *NRP1* | G | 0.17 | 1.06 (1.04-1.07) | Gormley et al., 2016 | Mean decrease accuracy < 0 |
| rs2223089 | 10 | Intergenic | near *ARMS2* | C | 0.08 | 0.93 (0.91-0.95) | Gormley et al., 2016 | Included |
| rs4910165 | 11 | Genic | *MRVI1* | C | 0.33 | 0.94 (0.91-0.98) | Gormley et al., 2016 | Included |
| rs10895275 | 11 | Genic | *YAP1* | A | 0.33 | 1.04 (1.03-1.06) | Gormley et al., 2016 | Mean decrease accuracy < 0 |
| rs561561 | 11 | Genic | *IGSF9B* | T | 0.12 | 0.94 (0.92-0.96) | Gormley et al., 2016 | Mean decrease accuracy < 0 |
| rs11031122 | 11 | Genic | *MPPED2* | C | 0.24 | 1.04 (1.03-1.06) | Gormley et al., 2016 | Mean decrease accuracy < 0 |
| rs11172113 | 12 | Intergenic | near *LRP1* | C | 0.42 | 0.90 (0.89-0.91) | Gormley et al., 2016 | Included |
| rs1024905 | 12 | Intergenic | near *FGF6* | G | 0.47 | 1.06 (1.04-1.08) | Gormley et al., 2016 | Mean decrease accuracy < 0 |
| rs11624776 | 14 | Intergenic | near *ITPK1* | C | 0.31 | 0.96 (0.94-0.97) | Gormley et al., 2016 | Included |
| rs8046696* | 16 | Genic | *CFDP1* | T | 0.45 | 1.05 (1.03-1.06) | Gormley et al., 2016 | Mean decrease accuracy < 0 |
| rs4081947 | 16 | Intergenic | near *ZCCHC14* | G | 0.34 | 1.03 (1.00-1.06) | Gormley et al., 2016 | Included |
| rs17857135 | 17 | Genic | *RNF213* | C | 0.17 | 1.06 (1.04-1.08) | Gormley et al., 2016 | Mean decrease accuracy < 0 |
| rs75213074 | 17 | Intergenic | near *WSCD1* | T | 0.03 | 0.89 (0.86-0.93) | Gormley et al., 2016 | Included |
| rs4814864 | 20 | Genic | *SLC24A3* | C | 0.26 | 1.07 (1.06-1.09) | Gormley et al., 2016 | Included |
| rs144017103 | 20 | Intergenic | near *CCM2L* | T | 0.02 | 0.85 (0.76-0.96) | Gormley et al., 2016 | Mean decrease accuracy < 0 |
| rs111404218 | 20 | Intergenic | near *JAG1* | G | 0.34 | 1.05 (1.03-1.07) | Gormley et al., 2016 | NA |
| rs12845494 | X | Intergenic | near *MED14* | G | 0.27 | 0.96 (0.95-0.97) | Gormley et al., 2016 | NA |

* In the meta-analysis by Gormley et al., (2016) the association is reported for rs77505915 (merged into rs8046696) and for rs140002913 (merged into rs9267918). Chr, chromosome; CI, confidence interval; GRS, genetic risk score; HWE, Hardy-Weinberg equilibrium; LD, linkage disequilibrium; MA, minor allele; MAF, minor allele frequency; NA, not available; OR, odds ratio; SNP, single nucleotide polymorphism.
