# Supplementary material for: A genetic risk score is differentially associated with migraine with and without aura
Source: Hum Genet. 2017 Jun 27;136(8):999–1008. doi: 10.1007/s00439-017-1816-5 (PMC5502071; doi:10.1007/s00439-017-1816-5)
Supplement: Supplementary file 2 — Supplementary material 2 (DOCX 26 kb) [file 439_2017_1816_MOESM2_ESM.docx]

**Supplementary table 2. Association analyses between selected genetic variants and migraine/migraine subtypes.**

|  |  |  | **Migraine (n = 446)** | | | | **MWA (N = 152)** | | | | **MWOA (N = 294)** | | | |
| --- | --- | --- | --- | --- | --- | --- | --- | --- | --- | --- | --- | --- | --- | --- |
|  |  |  | **vs controls (2,511)** | | | | | | | | | | | |
| **SNP** | **Gene** | **MA** | **OR** | **P** | **Adj OR^a^** | **Adj P^a^** | **OR** | **P** | **Adj OR^a^** | **Adj P^a^** | **OR** | **P** | **Adj OR^a^** | **Adj P^a^** |
| rs186166891 | *SUGCT* | T | 1.48 | **0.0004** | **1.54** | **0.0002** | 1.28 | 0.20 | 1.32 | 0.15 | **1.58** | **0.0004** | **1.66** | **0.0001** |
| rs4814864 | *SLC24A3* | C | 1.32 | **0.0008** | **1.30** | **0.0017** | **1.41** | **0.008** | **1.37** | **0.015** | **1.27** | **0.017** | **1.26** | **0.021** |
| rs1024905 | near *FGF6* | G | 1.19 | **0.016** | **1.20** | **0.018** | 0.86 | 0.20 | 0.86 | 0.20 | **1.41** | **0.0001** | **1.42** | **0.0001** |
| rs10218452 | *PRDM16* | G | 1.16 | 0.07 | **1.19** | **0.038** | 1.15 | 0.29 | 1.17 | 0.23 | 1.17 | 0.11 | 1.20 | 0.07 |
| rs28455731 | near *GJA1* | T | 1.16 | 0.15 | 1.17 | 0.11 | 1.21 | 0.25 | 1.24 | 0.19 | 1.13 | 0.31 | 1.15 | 0.27 |
| rs144017103 | near *CCM2L* | T | 0.64 | 0.18 | 0.60 | 0.13 | 0.37 | 0.17 | 0.34 | 0.13 | 0.77 | 0.49 | 0.74 | 0.42 |
| rs13208321 | *FHL5* | A | 1.11 | 0.19 | 1.15 | 0.11 | 1.12 | 0.38 | 1.13 | 0.37 | 1.11 | 0.30 | 1.15 | 0.16 |
| rs11624776 | near *ITPK1* | C | 0.91 | 0.25 | 0.91 | 0.23 | 0.98 | 0.87 | 0.97 | 0.81 | 0.88 | 0.18 | 0.88 | 0.17 |
| rs9349379 | *PHACTR1* | G | 0.93 | 0.32 | 0.93 | 0.38 | 1.02 | 0.85 | 1.02 | 0.85 | 0.88 | 0.17 | 0.90 | 0.21 |
| rs9267918* | near *NOTCH4* | A | 0.88 | 0.41 | 0.92 | 0.58 | 0.68 | 0.08 | 0.70 | 0.11 | 1.04 | 0.84 | 1.09 | 0.67 |
| rs10456100 | *KCNK5* | T | 1.07 | 0.43 | 1.09 | 0.32 | 0.89 | 0.41 | 0.90 | 0.44 | 1.17 | 0.12 | 1.19 | 0.08 |
| rs4081947 | near *ZCCHC14* | G | 1.06 | 0.44 | 1.07 | 0.40 | 0.89 | 0.37 | 0.90 | 0.38 | 1.16 | 0.11 | 1.18 | 0.09 |
| rs561561 | *IGSF9B* | T | 0.92 | 0.45 | 0.94 | 0.57 | 0.79 | 0.24 | 0.81 | 0.28 | 0.98 | 0.90 | 1.01 | 0.97 |
| rs17862920 | *TRPM8* | T | 0.93 | 0.49 | 0.92 | 0.49 | 1.12 | 0.52 | 1.14 | 0.44 | 0.83 | 0.19 | 0.82 | 0.17 |
| rs10915437 | near *AJAP1* | G | 0.95 | 0.49 | 0.95 | 0.55 | 1.11 | 0.40 | 1.11 | 0.42 | 0.87 | 0.14 | 0.89 | 0.18 |
| rs7577262 | *TRPM8* | A | 0.93 | 0.49 | 0.92 | 0.49 | 1.12 | 0.52 | 1.14 | 0.44 | 0.83 | 0.19 | 0.82 | 0.17 |
| rs11031122 | *MPPED2* | C | 1.06 | 0.50 | 1.03 | 0.77 | 0.85 | 0.23 | 0.83 | 0.20 | 1.18 | 0.09 | 1.13 | 0.21 |
| rs10504861 | near *MMP16* | T | 0.94 | 0.50 | 0.94 | 0.52 | 1.10 | 0.53 | 1.09 | 0.55 | 0.86 | 0.20 | 0.86 | 0.21 |
| rs1268083 | *LOC105377986* | C | 0.95 | 0.51 | 0.95 | 0.52 | 0.91 | 0.45 | 0.91 | 0.45 | 0.97 | 0.77 | 0.97 | 0.77 |
| rs10786156 | *PLCE1* | G | 0.95 | 0.52 | 0.95 | 0.50 | 0.91 | 0.40 | 0.90 | 0.39 | 0.98 | 0.82 | 0.98 | 0.77 |
| rs2274316 | *MEF2D* | C | 1.05 | 0.55 | 1.04 | 0.63 | 1.03 | 0.82 | 1.03 | 0.82 | 1.06 | 0.55 | 1.05 | 0.64 |
| rs6478241 | *ASTN2* | A | 0.96 | 0.58 | 0.95 | 0.49 | 0.92 | 0.52 | 0.93 | 0.58 | 0.98 | 0.80 | 0.97 | 0.71 |
| rs75213074 | near *WSCD1* | T | 1.12 | 0.61 | 1.14 | 0.55 | 0.84 | 0.65 | 0.83 | 0.65 | 1.26 | 0.35 | 1.30 | 0.30 |
| rs10155855 | near *DOCK4* | T | 0.96 | 0.68 | 0.93 | 0.63 | 1.10 | 0.68 | 1.10 | 0.69 | 0.88 | 0.51 | 0.84 | 0.37 |
| rs6790925 | near *TGFBR2* | T | 1.03 | 0.68 | 1.02 | 0.80 | 0.84 | 0.18 | 0.82 | 0.13 | 1.14 | 0.14 | 1.14 | 0.17 |
| rs17857135 | *RNF213* | C | 1.04 | 0.71 | 1,09 | 0,43 | 0.90 | 0.54 | 0.94 | 0.71 | 1.11 | 0.37 | 1.16 | 0.23 |
| rs11172113 | near *LRP1* | C | 0.97 | 0.71 | 0.98 | 0.82 | 0.80 | 0.07 | 0.79 | 0.06 | 1.08 | 0.41 | 1.10 | 0.28 |
| rs12260159 | *HPSE2* | A | 1.04 | 0.73 | 1.05 | 0.67 | 1.21 | 0.30 | 1.24 | 0.26 | 0.96 | 0.77 | 0.97 | 0.83 |
| rs10166942 | near *TRPM8* | C | 0.97 | 0.74 | 0.97 | 0.64 | 1.10 | 0.52 | 1.12 | 0.45 | 0.91 | 0.38 | 0.90 | 0.36 |
| rs10895275 | *YAP1* | A | 1.02 | 0.80 | 1.04 | 0.58 | 1.04 | 0.75 | 1.08 | 0.55 | 1.01 | 0.92 | 1.03 | 0.78 |
| rs2078371 | near *TSPAN2* | C | 1.03 | 0.82 | 1.05 | 0.71 | 0.96 | 0.82 | 0.98 | 0.93 | 1.06 | 0.65 | 1.07 | 0.64 |
| rs4910165 | *MRVI1* | C | 1.02 | 0.83 | 1.03 | 0.68 | 1.18 | 0.17 | 1.12 | 0.13 | 0.94 | 0.48 | 0.95 | 0.58 |
| rs7684253 | near *REST* | C | 1.02 | 0.84 | 1.01 | 0.94 | 0.84 | 0.15 | 0.85 | 0.19 | 1.12 | 0.20 | 1.11 | 0.27 |
| rs8046696* | *CFDP1* | T | 1.01 | 0.85 | 1.02 | 0.80 | 0.98 | 0.87 | 0.99 | 0.94 | 1.03 | 0.72 | 1.04 | 0.70 |
| rs2651899 | *PRDM16* | C | 1.01 | 0.85 | 1.02 | 0.78 | 0.94 | 0.59 | 0.95 | 0.64 | 1.06 | 0.53 | 1.07 | 0.47 |
| rs2506142 | *NRP1* | G | 1.01 | 0.89 | 1.01 | 0.95 | 1.09 | 0.58 | 1.07 | 0.67 | 0.98 | 0.83 | 0.97 | 0.80 |
| rs13078967 | near *GPR149* | C | 0.97 | 0.90 | 0.92 | 0.77 | 0.66 | 0.42 | 0.63 | 0.37 | 1.13 | 0.69 | 1.10 | 0.76 |
| rs1572668 | *1p31.1* | G | 1.01 | 0.90 | 1.02 | 0.80 | 1.03 | 0.80 | 1.04 | 0.75 | 0.99 | 0.99 | 1.00 | 0.98 |
| rs138556413 | *CARF* | T | 0.98 | 0.91 | 0.99 | 0.94 | 0.89 | 0.73 | 0.90 | 0.75 | 1.02 | 0.92 | 1.04 | 0.88 |
| rs2223089 | near *ARMS2* | C | 1.01 | 0.97 | 1.04 | 0.80 | 1.05 | 0.83 | 1.10 | 0.68 | 0.98 | 0.93 | 1.01 | 0.97 |

* In the meta-analysis by Gormley et al., (2016), the association is reported for rs77505915 (merged into rs8046696) and for rs140002913 (merged into rs9267918). The association between genetic variants and migraine/migraine subtypes was tested using logistic regression analyses.
**^a^**Analyses adjusted for age, sex and a lifetime diagnosis of major depressive disorder.

Adj, adjusted; MA, minor allele; MWA, migraine with aura; MWOA, migraine without aura; OR, odds ratio; SNP, single nucleotide polymorphism.
